# Evaluation of associations between estimates of particulate matter exposure and new onset type 2 diabetes in the REGARDS cohort

**DOI:** 10.1038/s41370-021-00391-9

**Published:** 2021-10-16

**Authors:** Tara P. McAlexander, S. Shanika A. De Silva, Melissa A. Meeker, D. Leann Long, Leslie A. McClure

**Affiliations:** 1 Department of Epidemiology and Biostatistics, Drexel University Dornsife School of Public Health, Philadelphia, PA USA; 2 Department of Biostatistics, University of Alabama at Birmingham School of Public Health, Birmingham, AL USA

**Keywords:** Air pollution, Diabetes, Particulate matter, Exposure assignment, Community type

## Abstract

**Background:**

Studies of PM_2.5_ and type 2 diabetes employ differing methods for exposure assignment, which could explain inconsistencies in this growing literature. We hypothesized associations between PM_2.5_ and new onset type 2 diabetes would differ by PM_2.5_ exposure data source, duration, and community type.

**Methods:**

We identified participants of the US-based REasons for Geographic and Racial Differences in Stroke (REGARDS) cohort who were free of diabetes at baseline (2003–2007); were geocoded at their residence; and had follow-up diabetes information. We assigned PM_2.5_ exposure estimates to participants for periods of 1 year prior to baseline using three data sources, and 2 years prior to baseline for two of these data sources. We evaluated adjusted odds of new onset diabetes per 5 µg/m^3^ increases in PM_2.5_ using generalized estimating equations with a binomial distribution and logit link, stratified by community type.

**Results:**

Among 11,208 participants, 1,409 (12.6%) had diabetes at follow-up. We observed no associations between PM_2.5_ and diabetes in higher and lower density urban communities, but within suburban/small town and rural communities, increases of 5 µg/m^3^ PM_2.5_ for 2 years (Downscaler model) were associated with diabetes (OR [95% CI] = 1.65 [1.09, 2.51], 1.56 [1.03, 2.36], respectively). Associations were consistent in direction and magnitude for all three PM_2.5_ sources evaluated.

**Significance:**

1- and 2-year durations of PM_2.5_ exposure estimates were associated with higher odds of incident diabetes in suburban/small town and rural communities, regardless of exposure data source. Associations within urban communities might be obfuscated by place-based confounding.

## Introduction

Particulate matter with a diameter ≤2.5 microns (PM_2.5_) is a ubiquitous ambient air pollutant with documented negative impacts on human health in epidemiologic studies [1, 2]. Mechanisms underlying documented associations between PM_2.5_ and health impacts include induction of oxidative stress, systemic inflammation, and endothelial dysfunction [2]. There is biologic rationale for associations between ambient PM_2.5_ exposures and the development of type 2 diabetes [2]. Specifically, systemic inflammation [3] and consequential metabolic dysfunction [4] are directly related to the development of type 2 diabetes [5–7]. Indirectly, exposure to PM_2.5_ can increase blood pressure and exacerbate hypertension [8], which is known to contribute to the development of type 2 diabetes [9].

While epidemiologic studies have extensively evaluated associations between PM_2.5_ and cardiovascular and respiratory disease and found consistent adverse associations, studies of the associations between PM_2.5_ and type 2 diabetes are less prevalent and demonstrate mixed results [10–16]. Although an increasing number of epidemiologic studies have found positive associations between PM_2.5_ and traffic-related PM exposures and type 2 diabetes outcomes [10–12, 15, 17–19], other robust epidemiology studies have found null associations [13, 14, 20, 21]. Inconsistencies in findings could be due to differences in PM_2.5_ composition and estimation by community types and regions; by population differences; and by exposure assignment choices. These decisions are a challenge in the epidemiology of PM_2.5_ and type 2 diabetes and include: the exposure model used (i.e., monitor-dependent, emissions-based, satellite-derived); the exposure duration and latency period assigned prior to diabetes outcome assessment [22]; the consideration of confounders (temperature, proximity to roadways, and co-pollutants such as ozone and oxides of nitrogen [NO_x_]); and the consideration of the chemical constituents of PM_2.5_.

Another challenge in understanding the epidemiology of PM_2.5_ and type 2 diabetes is the ability to adequately account for multiple risk factors for diabetes onset that occur at the community level (e.g., neighborhood walkability, healthy food access, availability of recreational spaces, traffic-related pollutants) [23, 24]. Often, these risk factors cluster within distinct community typologies [25–27]. Numerous studies have demonstrated that PM_2.5_ levels are generally higher in cities than in rural areas and correlate with proximity to roadways [28, 29], thus complicating the epidemiologic evaluation of PM_2.5_ and type 2 diabetes. Stratifying analyses of PM_2.5_ and type 2 diabetes by distinct community types is a computationally simple strategy to mitigate place-based confounding; however, many studies of PM_2.5_ and type 2 diabetes across diverse geographies did not consider community type as a potential confounder.

The goal of this study is to evaluate the extent to which different exposure assignment choices and PM_2.5_ data sources impact these associations and the extent to which community type modifies the associations between PM_2.5_ and type 2 diabetes. We hypothesize that PM_2.5_ exposure estimates for participants in the REasons for Geographic And Racial Differences in Stroke (REGARDS) cohort will differ by the data source used and by the duration of exposures assigned prior to type 2 diabetes onset, which would lead to differences in estimated odds of diabetes. Second, we hypothesize that a census tract-level measure of community type (e.g., higher density urban, lower density urban, suburban/small town, and rural) will modify associations between PM_2.5_ and diabetes.

## Methods

### Study population

Our analysis included participants from the REGARDS cohort. REGARDS is an observational study of risk factors for stroke in Black and white adults aged 45 years or older across the contiguous United States (US), with oversampling in the Southeastern US. Detailed study methods are published elsewhere [30]. Briefly, participants were selected from commercially available lists of residents and were recruited through a combination of mail notification and phone contact. Computer assisted phone interviews (CATI) were used to collect verbal consent and baseline risk factors, including demographics, smoking history, and cardiovascular risk factors. An in-home visit was then completed to collect blood pressure, blood and urine samples, and a signed informed consent, among other data. Follow-up phone contacts occur every six months to ascertain stroke events. A second extended CATI and in-home visit occurred ~10 years after the baseline, with similar data collected. The study is monitored and approved by institutional review boards at all participating institutions.

Participants included in this analysis had geocoded residential address information, were free from diabetes at baseline assessment (occurring in 2003–2007) and had observed diabetes status at a second in-home visit with blood glucose measurement and medication verification (occurring in 2013–2016). Diabetes was defined as: a fasting glucose measure of at least 126 mg/dL or a non-fasting glucose of at least 200 mg/dL, or the use of oral diabetes medications or insulin at the follow-up in-home exam.

### PM_2.5_ exposures

We obtained estimates of PM_2.5_ from three different data sources. The first was the Centers for Disease Control and Prevention Wide-ranging ONline Data for Epidemiologic Research (CDC WONDER) data [31], for which daily estimates of PM_2.5_ for the years 2003–2008 were available for REGARDS participants [32, 33]. This estimation method relied on an algorithm that incorporated air monitoring data from the United States Environmental Protection Agency Air Quality System (US EPA AQS) and satellite data from the National Aeronautics and Space Administration MODerate-resolution Imaging Spectroradiometer (NASA MODIS) estimation of Aerosol Optical Depth (AOD) [32]. Estimates were obtained for a 10 km × 10 km national grid, and participants were assigned the value for the grid containing their residential location [33]. We generated exposure estimates by calculating the mean of daily PM_2.5_ estimates, a common approach for estimating individual exposures [34], for 1-year prior to each participant’s baseline assessment using R for Statistical Computing [35] and Stata v.13 [36]. A small number of participants (*n* = 6) had a baseline interview date in early January of 2003, thus precluding the computation of exposure estimates since CDC WONDER data were not available prior to 2003. We also did not have exposure data assigned for an additional 5 individuals with baseline interviews in 2007, so we imputed estimates for a total of *n* = 11 (0.1%) individuals using mean imputation.

The second PM_2.5_ dataset we examined is publicly available from the CDC through the National Environmental Health Tracking Network [37] and uses the US EPA Downscaler model [38]. This model uses AQS monitor data as well as data from the Community Multiscale Air Quality (CMAQ) model to supplement in areas with sparse monitoring networks. Census tract-level estimates of PM_2.5_ are available daily for the years 2001–2014. We generated exposure estimates from the Downscaler model by calculating the annual mean daily PM_2.5_ estimates for the 1- and 2- years prior to the year of each participant’s baseline assessment.

Lastly, we obtained another publicly available PM_2.5_ dataset, global annual grid estimates provided by van Donkelaar et al. [39]. This source incorporated data from NASA MODIS, Multi-angle Imaging SpectroRadiometer (MISR) and Sea-viewing Wide Field-of-view Sensor (SeaWiFS) AOD data using geographically weighted regression to generate annual average estimates for the years 2000–2017, gridded at 0.01 degrees (~1.1 km). We downloaded annual raster datasets and calculated the average PM_2.5_ value within a 1-mile radius around each participant’s address using ArcGIS [40]. We assigned exposure estimates of: 1-year prior to baseline and the average of the two annual estimates for the 2-years prior to baseline.

### Covariates and community type definitions

Demographic characteristics and behaviors were assessed via the baseline CATI and included: age, gender (M/F), race (Black/white), smoking status (current, former, never), educational attainment (<high school, high school graduate, some college, ≥college graduate), and annual household income (<$20,000, $20,000–$34,000, $35,000–$74,000, ≥$75,000, refused to answer). Region was defined consistently with previous studies of this population (Stroke belt [Alabama, Arkansas, Louisiana, Mississippi, and Tennessee], buckle [North Carolina, South Carolina, and Georgia], non-belt [all other states in contiguous US]), identifying areas of higher stroke incidence in the Southeastern US [41]. Daily ambient temperature was estimated for REGARDS participants using the average of daily hourly data from the North American Land Data Assimilation System (NLDAS)[33]; we calculated annual average temperature for the year prior to the baseline assessment by averaging the daily values for each respective year.

Due to the potential for place-based confounding at the community level, we assigned each participant a community type (higher density urban, lower density urban, suburban/small town, and rural) for the census tract in which they resided. These classifications were derived from the US Department of Agriculture (USDA) Rural-Urban Commuting Area (RUCA) codes [42] and were modified to reflect the land area of each census tract based on the proportion of land area contained within a census-designated urbanized area or urban cluster, and by the size of tract land area [27].

### Statistical methods

We first computed descriptive statistics for all individual-level and community variables assigned to participants using Stata 13.1 [36] and stratified these by diabetes status and, separately, by community type. We compared distributions of these variables by diabetes status using analysis of variance (ANOVA) for continuous variables and Pearson’s χ^2^ tests for categorical variables. We calculated the mean PM_2.5_ exposures for each PM_2.5_ data source and duration, stratified by diabetes status, and we visualized the distributions of 1-year PM_2.5_ estimates for each of the three PM_2.5_ sources with histograms stratified by community type.

To evaluate our primary associations of PM_2.5_ estimates with the odds of new onset diabetes at follow-up, we used generalized estimating equations with a binomial distribution, a logit link function, an exchangeable correlation structure to account for clustering of individuals in census tracts, and robust standard errors. Models were stratified by community type and adjusted for the following covariates: age (centered and centered-squared), race, gender, annual income, region, smoking status, and annual average temperature in the year prior to baseline. We scaled estimates of each PM_2.5_ exposure to estimate the odds of new onset diabetes per 5 µg/m^3^ increase in PM_2.5_ exposure for ease of interpretation and relevance to the levels observed in this sample.

We conducted several sensitivity analyses to assess the robustness of our primary associations, including additional adjustment for year of enrollment and educational status, separately. We also evaluated models of CDC WONDER estimates that excluded the 11 individuals for whom we imputed PM_2.5_ estimates. To assess associations of shorter durations of PM_2.5_ exposures with new onset diabetes, we conducted sensitivity analyses using exposure durations of 2 weeks and 30 days prior to baseline assessment using the two data sources with daily estimates available (CDC Wonder and CDC Downscaler). To assess associations of longer durations of PM_2.5_ exposures with new onset diabetes, we conducted sensitivity analyses using the CDC Downscaler data for participants with a baseline enrollment date in 2004, 2005, 2006, or 2007 for exposure durations of 3 years (*n* = 9277) and for participants with an enrollment date in 2005, 2006, or 2007 for exposure durations of 4 years (*n* = 5961). We were unable to evaluate longer exposure durations in the full sample because CDC Downscaler data were not available prior to 2001; however, we did assess correlation between exposure durations of 1, 2, 3 and 4 years for participants with all exposure durations calculated (*n* = 5961).

## Results

Among the 11,208 participants free of diabetes at baseline, 1409 (12.6%) had type 2 diabetes at follow-up (Table 1). Compared to those without diabetes (*n* = 9799), individuals with diabetes were slightly younger (62.2 [SD: 7.8] vs. 63.2 [SD: 8.6]); individuals with diabetes were more often: Black individuals (46.3% vs. 30.8%), persons with annual income of <$20,000 (16.9% vs. 10.5%), and persons who currently smoke (15.4% vs. 10.5%). We did not observe any differences between community type and frequency of new onset diabetes (*p* = 0.7, Table 1). However, we did observe differences in some participant characteristics by community type (Table 2), including race, gender, educational attainment, annual income, smoking status, year of enrollment, region, and annual average temperature (*p* < 0.001 for each of these) and age (*p* = 0.009). These differences supported our a priori decision to stratify analyses of PM_2.5_ and new onset diabetes by community type.

**Table 1:** Baseline participant characteristics by diabetes status at follow-up.

	Diabetes status		*p* value^a^
Variable	No (*n* = 9799)	Yes (*n* = 1409)	
Age (mean, SD)	63.2 (8.6)	62.2 (7.8)	<0.001
Race (*n*, %)			
White	6777 (69.2)	757 (53.7)	<0.001
Black	3022 (30.8)	652 (46.3)	
Gender (*n*, %)			
Male	4297 (43.9)	655 (46.5)	0.06
Female	5502 (56.1)	754 (53.5)	
Education (*n*, %)			
<High school	603 (6.2)	163 (11.6)	<0.001
High school graduate	2169 (22.1)	278 (26.8)	
Some college	2521 (25.7)	400 (28.4)	
≥College graduate	4505 (46.0)	468 (33.2)	
Income (*n*, %)			
<$20,000	1032 (10.5)	238 (16.9)	<0.001
$20,000–$34,000	2044 (20.9)	339 (24.1)	
$35,000–$74,000	3342 (34.1)	474 (33.6)	
≥$75,000	2316 (23.6)	224 (15.9)	
Refused	1065 (10.9)	134 (9.5)	
Smoking status (*n*, %)			
Current	1029 (10.5)	216 (15.4)	<0.001
Past	3846 (39.4)	568 (40.5)	
Never	4893 (50.1)	619 (44.1)	
Community type (*n*, %)			
Higher density urban	1584 (16.2)	223 (15.8)	0.7
Lower density urban	3940 (40.2)	587 (41.2)	
Suburban/small town	1945 (19.9)	279 (19.8)	
Rural	2330 (23.8)	320 (22.7)	
Year of enrollment (*n*, %)			
2003	1642 (16.7)	286 (20.3)	0.002
2004	2883 (29.4)	435 (30.9)	
2005	2139 (21.8)	272 (19.3)	
2006	1690 (17.3)	214 (15.2)	
2007	1445 (14.8)	202 (14.3)	
Region (US Census) (*n*, %)			
Northeast	713 (7.3)	84 (6.0)	0.001
Midwest	1526 (15.4)	213 (15.1)	
South	6407 (65.4)	988 (70.1)	
West	1153 (11.8)	124 (8.8)	
Region (REGARDS) (*n*, %)			
Belt	713 (7.3)	84 (6.0)	0.06
Buckle	1526 (15.4)	213 (15.1)	
Nonbelt	6407 (65.4)	988 (70.1)	
Annual average temperature, °F, mean (SD)	61.8 (6.7)	61.5 (6.5)	<0.001

^a^For continuous variables, obtained from one-way ANOVA; for categorical variables, obtained from Pearson χ.

**Table 2:** Baseline participant characteristics by community type.

	Community type				
Variable	Higher density urban (*n* = 1807)	Lower density urban (*n* = 4527)	Suburban/small town (*n* = 2224)	Rural (*n* = 2650)	*p* value^a^
Age (mean, SD)	63.1 (8.6)	63.3 (8.7)	62.7 (8.4)	62.7 (8.2)	0.009
Race (*n*, %)					
White	716 (39.6)	2826 (62.4)	1779 (80.0)	2213 (83.5)	<0.001
Black	1091 (60.4)	1701 (37.6)	445 (20.0)	437 (16.5)	
Gender (*n*, %)					
Male	706 (39.1)	2049 (45.3)	1034 (46.5)	1163 (43.9)	<0.001
Female	1101 (60.9)	2478 (54.7)	1190 (53.5)	1487 (56.1)	
Diabetes status (*n*, %)					
No	1584 (87.7)	3940 (87.0)	1945 (87.5)	2330 (87.9)	0.7
Yes	223 (12.3)	587 (13.0)	279 (12.5)	320 (12.1)	
Education (*n*, %)					
<High school	146 (8.1)	244 (5.4)	144 (6.5)	232 (8.8)	<0.001
High school graduate	433 (24.0)	910 (20.1)	469 (21.1)	735 (27.7)	
Some college	442 (24.5)	1199 (26.5)	572 (25.7)	708 (26.7)	
≥College graduate	786 (43.5)	2173 (48.0)	1039 (46.7)	975 (36.7)	
Income (*n*, %)					
<$20,000	260 (14.4)	477 (10.5)	205 (9.2)	328 (12.4)	<0.001
$20,000–$34,000	427 (23.6)	931 (20.6)	396 (17.8)	629 (23.7)	
$35,000–$74,000	553 (30.6)	1592 (35.2)	772 (34.7)	899 (33.9)	
≥$75,000	367 (20.3)	1075 (23.8)	597 (26.8)	501 (18.9)	
Refused	200 (11.1)	452 (10.0)	254 (11.4)	293 (11.1)	
Smoking status (*n*, %)					
Current	242 (13.5)	494 (10.9)	217 (9.8)	292 (11.1)	<0.001
Past	749 (41.7)	1773 (39.3)	879 (39.7)	1013 (38.3)	
Never	805 (44.8)	2250 (49.8)	1120 (50.5)	1337 (50.6)	
Year of enrollment (*n*, %)					
2003	334 (18.5)	809 (17.9)	370 (16.6)	413 (15.7)	<0.001
2004	605 (33.5)	1414 (31.2)	615 (27.7)	684 (24.8)	
2005	373 (20.6)	949 (21.0)	519 (23.3)	570 (21.5)	
2006	282 (15.6)	730 (16.1)	361 (16.2)	531 (20.0)	
2007	213 (11.8)	625 (13.8)	359 (16.1)	450 (16.9)	
Region (REGARDS) (*n*, %)					
Belt	165 (9.1)	1646 (36.4)	839 (37.7)	1104 (41.7)	<0.001
Buckle	59 (3.3)	725 (16.0)	684 (30.8)	934 (35.2)	
Nonbelt	1583 (87.6)	2156 (47.6)	701 (31.5)	612 (23.1)	
Annual average temperature, °F, mean (SD)	56.9 (6.6)	61.5 (6.7)	61.9 (6.3)	61.8 (6.1)	<0.001

^a^For continuous variables, obtained from one-way ANOVA; for categorical variables, obtained from Pearson χ^2^.

**Fig. 1:**
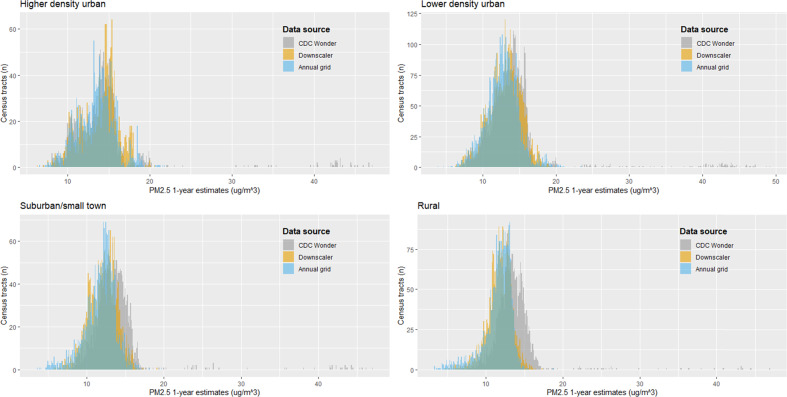
Histograms of annual PM_2.5_ exposure estimates for three sources, stratified by community type. Each panel illustrates the community type-specific distributions of 1-year estimates for the CDC WONDER data source, the Downscaler data source, and the Annual grid data source.

Within community type, the distributions of 1-year PM_2.5_ estimates were similar across sources, except for rural areas, where estimates from the CDC WONDER model were slightly higher than for the other two PM_2.5_ sources (Fig. 1). Mean 1-year PM_2.5_ estimates from all three sources differed by community type (*p* < 0.001), with highest mean values in higher density urban community types and lowest mean values in rural community types (Fig. 1). We also evaluated the differences in PM_2.5_ estimates by diabetes status for all sources and durations (Table 3), and we observed significantly higher mean long-term PM_2.5_ estimates (1- and 2-year) for participants who had diabetes compared to those who did not, although the magnitude of these differences was small.

**Table 3:** PM_2.5_ and constituent exposure, by source, duration, and diabetes status at follow-up.

	Diabetes status		
Variable	No (*n* = 9799)	Yes (*n* = 1409)	*p* value^a^
CDC WONDER PM_2.5_ (µg/m^3^, mean, SD)			
1-year duration	13.8 (4.1)	14.1 (4.2)	0.03
Downscaler PM_2.5_ (µg/m^3^, mean, SD)			
1-year duration	12.7 (2.1)	12.8 (2.1)	0.01
2-year duration	12.8 (2.2)	12.9 (2.1)	0.03
Annual grid PM_2.5_ (µg/m^3^, mean, SD)			
1-year duration	12.4 (2.2)	12.6 (2.1)	0.001
2-year duration	12.4 (2.2)	12.6 (2.1)	0.001

^a^Obtained from one-way ANOVA.

**Fig. 2:**
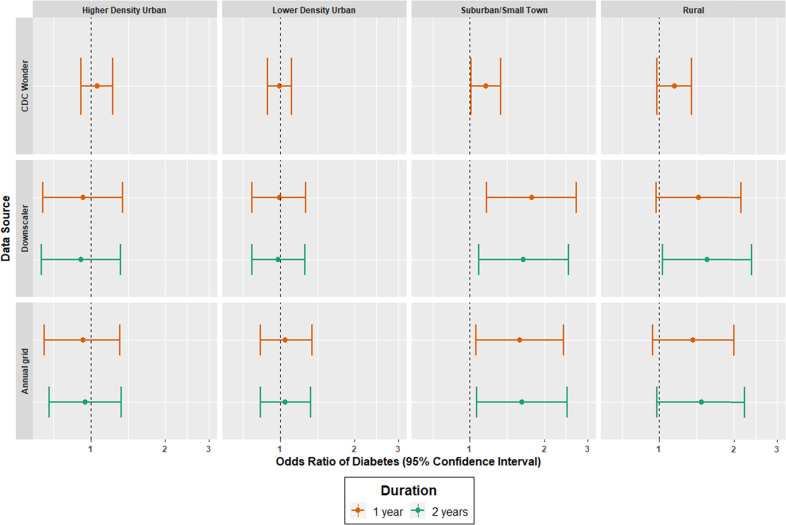
Forest plots of estimated odd ratios and 95% confidence interval of new onset diabetes per 5 ug/m^3^ increase of PM_2.5_, by community type, exposure source and duration. Models fitted using GEE with robust standard errors adjusted for age (centered and centered-squared), race, gender, income category, smoking status, annual average temperature, and region of REGARDS study, clustered on Census tract.

After adjusting for a priori defined covariates, we did not observe associations between any measure of PM_2.5_ exposure and incident diabetes within higher and lower density urban community types (Fig. 2). Within suburban/small town community types, odds of diabetes were higher per 5 µg/m^3^ increase in 1-year estimates of PM_2.5_ for each of the three sources evaluated (Fig. 2): CDC WONDER (OR [95% CI] per 5 µg/m^3^ increase in PM_2.5_: 1.16 [1.01, 1.33]), Downscaler (OR [95% CI] per 5 µg/m^3^ increase in PM_2.5_: 1.78 [1.17, 2.69]), annual grid (OR [95% CI] per 5 µg/m^3^ increase in PM_2.5_: 1.59 [1.06, 2.39]). We also observed significant associations with diabetes for the 2-year annual grid estimates in suburban/small towns: Downscaler (OR [95% CI] per 5 µg/m^3^ increase in PM_2.5_: 1.65 [1.09, 2.51]), and annual grid (OR [95% CI] per 5 µg/m^3^ increase in PM_2.5_: 1.62 [1.07, 2.48]). Within rural community types, the Downscaler and annual grid sources demonstrated trends of higher odds of diabetes with increasing duration of PM_2.5_ exposure; only the 2-year estimates of PM_2.5_ obtained from the Downscaler model were significantly associated with higher odds of diabetes: (OR [95% CI]) per 5 µg/m^3^ increase in PM_2.5_: 1.56 [1.03, 2.36], Fig. 2).

Sensitivity analyses for models with the additional adjustment for participants’ educational attainment or year of enrollment in REGARDS did not substantially or inferentially change our primary results, nor did the results of a model that excluded 11 individuals with imputed CDC WONDER estimates (results not shown). We did not observe any associations of PM_2.5_ with new onset type 2 diabetes when using shorter exposure durations of 2 weeks and 30 days prior to baseline enrollment for the CDC WONDER or Downscaler models (Supplementary Table S1). Among participants enrolled in 2005, 2006 and 2007 (*n* = 5961) for whom we were able to assign PM_2.5_ exposures of up to 4 years prior to baseline enrollment using the Downscaler model, Spearman correlation coefficients for longer exposure durations were ≥0.94 (Supplementary Table S2). We report only the effect estimates obtained from the sensitivity analysis of exposure durations of 3 years (*n* = 9277, Supplementary Table S3), as models with the 4-year exposure estimates (*n* = 5961) were unable to achieve convergence due to reduced sample size. Among the 9277 participants for whom we were able to assign a 3-year exposure duration with the Downscaler model, the magnitude of effect estimates was similar to the primary models (1 & 2 year durations); however, only the effect estimate within rural community types was statistically significant: (OR [95% CI] per 5 µg/m^3^ increase in PM_2.5_: 1.66 [1.03, 2.65]).

## Discussion

Exposure estimates of PM_2.5_ were associated with higher odds of new onset type 2 diabetes in this study of 11,208 participants from the REGARDS cohort residing in suburban/small town and rural community types; however, these associations were only observed for exposure durations of at least 1-year. Observed associations were similar regardless of the data source of the PM_2.5_ exposure estimates. We did not observe associations between PM_2.5_ exposure estimates in higher density or lower density urban community types. These findings suggest that differences in the association of PM_2.5_ and type 2 diabetes by community type might account for some of the heterogeneity in the strength and significance of associations between PM_2.5_ and diabetes outcomes reported in the epidemiologic literature to date [28].

We found that longer term (1-year and 2-year) durations of PM_2.5_ exposures were associated with type 2 diabetes. These are biologically plausible associations; development of type 2 diabetes is consistent with pathophysiologic mechanisms of systemic inflammation, dysfunction of insulin-producing β-cells, and glucose sensitivity associated with chronic PM_2.5_ exposures [28], so it is plausible that these associations were not present for the shorter-term exposure durations evaluated. It is also possible that the variation in shorter-term exposures may not capture the cumulative effects associated with a longer-term exposure. The magnitude of effect estimates observed for 5 µg/m^3^ increases in 1-year PM_2.5_ durations was also consistent with the sizes of effect estimates in other studies, though effect estimates within suburban/small towns from the Downscaler model and annual grid were approximately twice as large as the effect estimates commonly observed in the epidemiologic literature [17, 43, 44]. It is possible that effect estimates were stronger because of our community type stratification approach. While other studies [17, 45, 46] of air pollution and incident diabetes have conducted analyses stratified by important factors (e.g., individual-level risk factors, region, year, neighborhood-level socioeconomic status), we have not identified any studies that evaluated PM_2.5_ and incident diabetes in the US in a community type stratified approach.

We did not observe any associations between PM_2.5_ estimates and new onset diabetes within higher density and lower density urban community types; however, we did observe differences in mean PM_2.5_ estimates by community types in the direction that we expected, with higher and lower density urban community types having higher mean PM_2.5_ compared to suburban/small towns and rural areas. In addition to potential exposure misclassification that is differential with respect to community type, it is possible that within community types, there is place-based confounding by community-level factors that are related to community type as well as diabetes onset, such as neighborhood walkability, healthy food access, and opportunities for recreational physical activity [24–26] that may impact potential associations and are contextually relevant to an individual’s diabetes risk in urban vs. rural environments [47]. Future studies would need to carefully measure and evaluate these multidimensional and often overlapping community level factors that influence diabetes risk in addition to PM_2.5_ exposures.

We initially hypothesized that exposure estimates would differ depending on the method of exposure assessment used, and we expected the largest differences to be between exposure estimates from the annual grid, which were centered at the participants’ homes and estimates from the CDC WONDER and Downscaler models, which were estimated for participants’ census tracts. However, the distributions of these estimates and their values were relatively similar across sources and within community types, with an exception in rural community types, where estimates of PM_2.5_ from the CDC WONDER model were slightly larger than those from the Downscaler or annual grid models. The general concordance of estimates across methods gives us confidence in the accuracy of each exposure assessment method used and suggests that differing PM_2.5_ estimation methods are likely not the primary driver of mixed results in epidemiologic studies of PM_2.5_ and diabetes, although differing PM_2.5_ data sources not evaluated in this study could lead to conflicting results.

Our study is not without limitations. Primarily, we note that the exposure durations evaluated might not have been long enough to reflect chronic PM_2.5_ exposures relevant to diabetes risk. Mechanistically, it is very likely that new onset diabetes is a function of PM_2.5_ exposure of durations longer than 1 or 2 years. However, the availability and accuracy of historical PM_2.5_ data is a challenge [48], as are the limitations to historical residence information among participants in cohort studies [49]. Given these challenges, we believe the evaluation of durations of 1-year can be used to approximate long term exposure to PM_2.5_, and we observed high correlation among 1, 2, 3, and 4 year exposure estimates for a subset of individuals. We conclude that 1-year exposure durations are likely a sufficiently long enough exposure period for influencing diabetes risk in the years following, and that the 1-year measure likely serves as a good proxy for longer term exposure. Other limitations of this study include the potential for residual confounding by individual and community level factors not accounted for in our models. Further, we were unable to retrospectively understand participants’ behavior with respect to daily indoor and outdoor activities that would influence their individual exposure to PM_2.5_. Presumably, having personal air pollution monitor information for these participants would give us a better understanding of each participants’ actual PM_2.5_ exposure rather than what was assigned to their residential address.

There were also several strengths to this study. First, as our study sample was obtained from the REGARDS cohort, we had extensive survey and biometric health data from a large group of Black and white adults across the continental US. Although there have been other longitudinal studies of PM_2.5_ and type 2 diabetes, many studies have not been able to definitively exclude prevalent diabetes at baseline and therefore could not distinguish new onset diabetes from prevalent diabetes at follow-up; we were able to do so [28]. Another strength of this study is the examination of three differing exposure data sources to evaluate PM_2.5_, as each data source relied on slightly different methods (measurement and/or models) to estimate PM_2.5_ levels. However, estimates of PM_2.5_ and their associations with new onset type 2 diabetes were comparable across all three data sources evaluated.

This study adds support to the epidemiologic evidence that longer-term PM_2.5_ exposures are associated with diabetes risk. Our results also demonstrate that consideration of community type is important, although we suspect that place-based confounding was still present in our observed associations, particularly within the urban community types. We know that community factors such as healthy food availability and walkability are related to both place and to diabetes risk; we suspect that the epidemiologic relationships among these variables are also complex. As the epidemiology of PM_2.5_ exposures expands to implicate more adverse health conditions, studies that evaluate PM_2.5_ exposure should also consider the role of multiple, overlapping neighborhood level exposures that impact diabetes risk. Accounting for these exposures in epidemiologic studies necessitates careful evaluation of place-based clustering within the exposure data, and, if present, the implementation of sophisticated statistical methods to account for highly correlated exposure variables and better understand diabetes risk.

## Supplementary information

Supplemental Material
