# Supplementary material for: Evaluation of associations between estimates of particulate matter exposure and new onset type 2 diabetes in the REGARDS cohort
Source: J Expo Sci Environ Epidemiol. 2021 Oct 16;32(4):563–70. doi: 10.1038/s41370-021-00391-9 (PMC9012798; doi:10.1038/s41370-021-00391-9)
Supplement: Supplementary file 1 — Supplemental Material [file 41370_2021_391_MOESM1_ESM.docx]

**Table S1**. Estimated odd ratios and 95% confidence interval of new onset diabetes per 5 ug/m^3^ increase of PM_2.5_, by community type, exposure source and durations of 2 weeks and 30 days (n = 11,208). Models fitted using GEE with robust standard errors adjusted for age (centered and centered-squared), race, gender, income category, smoking status, annual average temperature, and region of REGARDS study, clustered on Census tract.

|  | **Odds Ratio (95 % CI)** | | | |
| --- | --- | --- | --- | --- |
| **Exposure source and duration** | **Higher density urban  (n = 1,807)** | **Lower density urban  (n = 4,527)** | **Suburban/small town (n = 2,224)** | **Rural (n = 2,650)** |
| **CDC WONDER**  2 weeks  30 days | 0.96 (0.81, 1.13)  0.99 (0.82, 1.20) | 0.96 (0.86, 1.06)  1.01 (0.89, 1.15) | 1.08 (0.92, 1.28)  1.12 (0.93, 1.35) | 1.09 (0.94, 1.27)  1.09 (0.91, 1.30) |
| **Downscaler**  2 weeks  30 days | 0.97 (0.81, 1.16)  0.98 (0.79, 1.21) | 0.93 (0.83, 1.04)  0.97 (0.84, 1.11) | 1.13 (0.94, 1.35) 1.15 (0.94, 1.41) | 1.13 (0.96, 1.34)  1.17 (0.96, 1.42) |

**Table S2.** Spearman correlation coefficients for Downscaler PM_2.5_ estimates with exposure durations of 1, 2, 3, and 4 years among participants enrolled in 2005, 2006 and 2007 (n = 5,961).

| **Duration** | **1 year** | **2 years** | **3 years** | **4 years** |
| --- | --- | --- | --- | --- |
| **1 year** | 1.00 |  |  |  |
| **2 years** | 0.98 | 1.00 |  |  |
| **3 years** | 0.96 | 0.99 | 1.00 |  |
| **4 years** | 0.94 | 0.98 | 0.99 | 1.00 |

**Table S3**. Estimated odd ratios and 95% confidence interval of new onset diabetes per 5 ug/m^3^ increase of PM_2.5_, by community type and exposure duration of 3 (n = 9,277) years from the Downscaler model. Model was fit using GEE with robust standard errors adjusted for age (centered and centered-squared), race, gender, income category, smoking status, annual average temperature, and region of REGARDS study, clustered on Census tract.

|  | **Odds Ratio (95 % CI)** | | | |
| --- | --- | --- | --- | --- |
| **Exposure source and duration** | **Higher density urban  (n = 1,471)** | **Lower density urban  (n = 3,717)** | **Suburban/small town (n = 1,854)** | **Rural (n = 2,235)** |
| **Downscaler**  3 years | 0.89 (0.60, 1.31) | 1.06 (0.81, 1.39) | 1.42 (0.93, 2.18) | 1.66 (1.03, 2.65) |
